# Trajectories of Life Course Financial Disadvantage and Depressive Mood: Results From the National Survey of the Japanese Elderly

**DOI:** 10.2188/jea.JE20250159

**Published:** 2026-04-05

**Authors:** Hiroshi Murayama, Erika Kobayashi, Hidehiro Sugisawa, Benjamin A. Shaw, Jersey Liang

**Affiliations:** 1 Research Team for Social Participation and Healthy Aging, Tokyo Metropolitan Institute for Geriatrics and Gerontology, Tokyo, Japan; 2 Graduate School of International Studies, J. F. Oberlin University, Tokyo, Japan; 3 Division of Community Health Sciences, University of Illinois, Chicago, IL USA; 4 University of Michigan School of Public Health, Ann Arbor, MI USA

**Keywords:** financial disadvantage, trajectories, life course, depressive mood, old age

## Abstract

**Background:**

Health status in old age can be influenced by financial disadvantages both at present and in earlier life stages; however, few studies have focused on the long-term individual patterns of financial disadvantage over the life course. This study examined the relationship between trajectories of financial disadvantage over the life course and depressive mood among community-dwelling older Japanese adults.

**Methods:**

Data were obtained from the 2012 National Survey of the Japanese Elderly using a two-stage stratified random sampling method. The sample consisted of 1,324 adults aged ≥60 years. We retrospectively assessed financial disadvantage at four life-course benchmark periods: ≤18 years old, 25–35 years old, 35–50 years old, and current age. Depressive mood was measured using the 8-item Center for Epidemiologic Studies Depression Scale.

**Results:**

We identified five distinct life-course financial disadvantage trajectories using group-based mixture modeling: “persistently affluent” (22.1%), “increasing affluence” (21.7%), “consistently modest” (28.0%), “decreasing affluence” (11.3%), and “persistently poor” (17.0%). A logistic regression analysis showed that people in the “increasing affluence” subgroup were less likely to have a depressive mood than those in the “persistently poor” subgroup, after adjusting for potential covariates, including current income and parental educational attainment. This association was more prominent in women than in men.

**Conclusion:**

The experience of escaping from financial disadvantages may bolster the mental health of older adults, regardless of sociodemographic characteristics, health behaviors, and health conditions. The mental health benefits of increasing affluence throughout the life course may be even stronger than the benefits of experiencing persistent affluence.

## INTRODUCTION

Depression is one of the most common mental illnesses worldwide.^1^ Experiencing depression later in life can increase the risks of mortality,^2^ dementia,^3^ frailty,^4^ and social isolation,^5^ so it is a pressing social issue and a major public health concern with broad implications. While clinical depression is a well-defined condition, depressive mood, which is characterized by a broader range of emotional distress, represents a critical but often overlooked aspect of mental health in aging populations. Among older adults, depressive mood is common and linked to functional decline^6^ and dementia.^7^ Understanding depressive mood is essential, as it impacts quality of life and may serve as an early indicator or precursor to more severe depressive disorders.

Numerous factors contribute to a depressive mood later in life. One critical determinant is socioeconomic status (SES) over the life course.^8^^,^^9^ Persistent or recurrent financial disadvantages exacerbate stress and limit access to resources supporting mental well-being.^10^^,^^11^ A life-course perspective provides a valuable framework for understanding how socioeconomic conditions throughout childhood and adulthood shape mental health outcomes during older ages. Several causal models have been proposed to explain the links between life-course SES and health outcomes, such as the pathway, accumulation, and latent period models.^12^ While distinguishing between these models empirically is difficult,^13^ they each suggest that social mobility over the life course is critical to understanding how socioeconomic factors across a person’s lifespan influence health outcomes.

Previous studies focused on social mobility and later-life health often use a priori classifications based on SES measures at multiple time points. For example, Szanton et al^14^ combined dichotomized variables of childhood and adult financial strain to create a life-course financial strain variable (ie, high-high, high-low, low-high, or low-low). They found that people with childhood and adulthood financial strain and only adulthood strain had higher probabilities of depressive symptoms than people with no strain in their life course, while those with only childhood strain were less likely to be depressed. This is an important finding, but given that socioeconomic conditions are likely to fluctuate substantially over the life course, this type of prespecified (or crude) classification relying on only two time points may fail to capture the more complex and dynamic nature of SES. Thus, tracking long-term individual patterns of change over time in SES—trajectories of socioeconomic conditions—may provide important additional information.

In this context, group-based mixture modeling (GBMM) offers a useful data-driven alternative to identify latent subgroups following similar longitudinal patterns of SES changes without arbitrary cut-offs or researcher-defined categories. Compared to prespecified classifications, GBMM reduces potential bias by allowing the data itself to determine the number and shape of trajectories. Prior literature notes that prespecified groupings can obscure meaningful within-group variation or fail to detect nonlinear or irregular patterns in SES changes over time.^15^ Additionally, we can better capture the dynamic and heterogeneous nature of socioeconomic trajectories and their associations with mental health outcomes. Previous studies have investigated the link between the patterns of life-course trajectories in SES and health outcomes, such as functional disability,^16^ physical function,^16^^,^^17^ cognitive function/brain atrophy,^16^^,^^18^ comorbidities,^16^^,^^17^ and mortality.^19^ However, the life-course trajectories of financial disadvantages and their specific relationship with depressive mood in older adults remain underexplored.

A United States study identified eight income trajectories over 25 years among men aged 25–49 years, including stable (low, middle, and high), increasing, and declining patterns.^20^ It found that income stability mattered more for mental health than absolute income—men with stable middle-income trajectories had similar mental health to those with high income. While informative, this study focused on younger adult men in a Western context and did not address older populations. Further research is needed to examine how long-term financial disadvantage affects depressive mood in older adults, particularly in non-Western settings.

Given these considerations, this study aimed to examine the relationship between trajectories of financial disadvantage over the life course and depressive mood among community-dwelling older Japanese adults. By leveraging longitudinal data, this study sought to identify long-term patterns of financial hardship and their corresponding impacts on depressive mood in later life. GBMM was well suited to our study, as it models distinct trajectories of financial disadvantage across multiple time points without imposing assumptions regarding number or shape. The Japanese context, with its rapidly aging population and unique social and cultural system, offers a critical lens through which these dynamics can be examined. Sugisawa et al^11^ examined the relationship between life-course financial strain and health outcomes, including depressive mood; however, they applied an a priori classification of changes in financial strain and failed to consider the dynamic nature of long-term changes in financial strain. Understanding how financial trajectories influence depressive mood in older adults in Japan may provide insights applicable globally.

## METHODS

### Participants

Data were obtained from the 2012 National Survey of the Japanese Elderly (NSJE), an ongoing nationwide longitudinal survey of community-dwelling Japanese adults aged ≥60 years. The first sample was collected in 1987 and supplemented in 1990, 1996, 1999, 2012, and 2021. Trained interviewers conducted face-to-face interviews in participants’ homes. This study uses cross-sectional data from the 2012 survey (*n* = 2,500). A two-stage stratified random sampling method was used, with stratification by geographic region and municipal population size. In the first stage, 192 census enumeration districts were selected; in the second, 2,500 individuals aged 60–92 years were randomly selected from the basic resident registers of these districts. Further details on the survey methodology are available elsewhere.^21^ Responses obtained from proxy interviews were excluded. Consequently, 1,324 participants completed the survey (response rate: 53.0%). The NSJE protocol was approved by the Institutional Review Board of the Tokyo Metropolitan Institute for Geriatrics and Gerontology.

### Measures

#### Financial disadvantage over the life course

Previous studies have retrospectively examined financial disadvantages in childhood, young adulthood, and middle age, reporting associations with health conditions in older age.^10^^,^^14^^,^^22^^,^^23^ Therefore, this study evaluated the life-course financial disadvantages using retrospective self-reported financial-strain indicators. We set four benchmark periods in the life course: ≤18 years old (childhood), 25–35 years old (young adults), 35–50 years old (middle age), and current age (old age). Financial strain in the ≤18 years old period was assessed using an item: “Did your family have difficulties covering expenditures for necessities, such as food, clothes, and housing?”. Respondents answered using a four-point Likert scale (1 = “very difficult,” 2 = “somewhat difficult,” 3 = “little difficult,” or 4 = “completely not difficult”). Regarding the questions for the 25–35 and 35–50 years old periods, we changed the term “your family” to “you” (ie, “Did you have trouble covering expenditures for necessities, such as food, clothes, and housing?”). Finally, current financial strain (ie, that in old age) was assessed by asking “How do you rate your family’s household finances?” and assessed using a five-point Likert scale (1 = “very difficult,” 2 = “somewhat difficult,” 3 = “neither,” 4 = “little difficult,” or 5 = “completely not difficult”). To ensure the comparability of each measure of financial status, we mathematically adjusted the score range of the current financial strain from 1–5 to 1–4 for our analysis (ie, original scores of 1, 2, 3, 4, and 5 were linearly transformed to 1.00, 1.75, 2.50, 3.25, and 4.00, respectively).

#### Depressive mood

We used the 8-item Center for Epidemiologic Studies Depression (CES-D-8) Scale to assess depressive mood.^24^ Respondents answered using a four-point Likert scale (0 = “rarely or none of the time,” 1 = “some or a little of the time [1–2 days],” 2 = “occasionally or a moderate amount of time [3–4 days],” or 3 = “all of the time [5–7 days]”), and the answers were summed (score range: 0–24). The Cronbach’s alpha in this study was 0.70. A cut-off point of 8/9 was used in the analysis, with a score of ≥9 indicating depressive mood.^25^

#### Covariates

Sociodemographic factors included age, sex, marital status (“married,” “never married,” “divorced,” or “widowed”), living alone (“yes” or “no”), employment status (“currently working” or “not working”), years of education (“≤9 years,” “10–12 years,” or “≥13 years”), and annual individual/couple income (“≤2.9 million yen,” “3.0–3.9 million yen,” or “≥4.0 million yen”). The average income of older adult households in 2010 in Japan was approximately 3.1 million yen, with 50% of the population earning <3.0 million yen, and the top 25% >4.0 million yen.^26^

Health behaviors and health conditions included smoking habit (“currently smoking” or “not smoking”), exercise habit (“often/sometimes” or “rarely/none”), disease possession, functional capacity, cognitive impairment, and a feeling of loneliness. Disease possession included hypertension, heart disease, cerebrovascular disease, cancer, and diabetes mellitus. Functional capacity entailed the sum of difficulties with six basic activities of daily living (dressing, walking, bathing or showering, eating, getting in or out of bed, and using the toilet) and five instrumental activities of daily living (grocery shopping, phone calls, climbing stairs, walking a few blocks, and traveling by bus or train). All items were scored on a five-point scale (1 = “unable to do” to 5 = “no difficulty”). The answers were summed (score range: 11–55). The Cronbach’s alpha was 0.92. Lower scores reflect greater functional disability.

Cognitive impairment was assessed using Pfeiffer’s Short Portable Mental Status Questionnaire, a 10-item screening instrument.^27^ In the NSJE, we used nine items, excluding questions about the participants’ telephone numbers. The score ranges from 0 to 9, with a higher score indicating more severe cognitive impairment. Finally, we measured a feeling of loneliness using a single item, “How often do you feel that you are isolated from others?”. Respondents answered with three options (“often,” “sometimes,” or “rarely”).

In addition to life-course financial disadvantage, we assessed parental educational attainment. Low parental education has often been used as an indicator of childhood adversity.^28^ We separately asked about the highest level of education attained by the respondent’s father and mother: “no formal education,” “junior high school graduation,” “high school graduation,” or “university graduation.”

### Statistical analysis

First, we used the GBMM to identify the distinct trajectories of financial disadvantage.^29^ We estimated models with two to eight trajectories by assuming linear, quadratic, and cubic patterns of change in financial disadvantage over the life course. We selected the best-fitting model (ie, the number of distinct trajectories) by comparing the Akaike Information Criterion (AIC) and Bayesian Information Criterion (BIC) scores associated with various solutions and the average posterior probabilities of group membership and by evaluating whether successive models identify additional distinct groups, as indicated by non-overlapping 95% confidence intervals (CIs).^30^

Second, binary logistic regression analysis was performed to examine the relationship between the trajectories of life-course financial disadvantage and depressive mood. We adjusted for age and sex in model 1 and controlled for the other covariates in addition to age and sex in model 2. The results were presented as odds ratios (ORs) with 95% CIs, and the predicted probabilities of depressive mood were calculated based on model 2. To understand the potential heterogeneities, we conducted stratified analyses by age group (60–74 years and ≥75 years) and sex. All analyses were performed using STATA 17 (StataCorp LLC, College Station, TX, USA).

Multiple imputations for missing data were employed to minimize the potential bias associated with item non-response. In particular, 20 complete datasets were imputed, and analyses were run on each of these 20 datasets, with parameter estimates derived by averaging across 20 imputations and adjusting for their variance. All analyses were conducted using imputed datasets.

## RESULTS

Table 1 shows the characteristics of the 1,324 participants. The mean age was 71.3 (standard deviation, 7.7) years, and 49.1% were men. A quarter (26.1%) had ≥13 years of education, and approximately half (49.1%) of the participants had an annual income of ≤2.9 million yen. The proportion of those with a score of ≥9 in the CES-D-8 (ie, those with depressive mood) was 17.1%.

**Table 1.** Participants’ characteristics (*n* = 1,324)

		Non-imputed	Imputed
Age, years		71.3 (7.7)	71.3 (7.7)
	Missing	0.0	
Sex	Men	49.1	49.1
	Missing	0.0	
Marital status	Married	74.2	74.2
	Never married	2.6	2.6
	Divorced	4.5	4.5
	Widowed	18.7	18.7
	Missing	0.0	
Living alone		12.8	12.8
	Missing	0.0	
Employment status	Currently working	36.1	36.1
	Missing	0.0	
Years of education	≤9 years	31.7	31.7
	10–12 years	42.1	42.1
	≥13 years	26.1	26.1
	Missing	0.0	
Annual individual/couple income	≤2.9 million yen	41.7	49.1
	3.0–3.9 million yen	19.9	23.4
	≥4.0 million yen	22.7	27.5
	Missing	15.7	
Smoking habit	Currently smoking	14.2	14.2
	Missing	0.2	
Exercise habit	Often/sometimes	65.2	65.3
	Missing	0.2	
Hypertension		41.7	42.0
	Missing	0.5	
Heart disease		11.8	12.0
	Missing	1.4	
Cerebrovascular disease		5.4	5.6
	Missing	0.9	
Cancer		5.0	5.3
	Missing	1.4	
Diabetes mellitus		12.4	12.6
	Missing	1.0	
Functional capacity (score range: 11–55)		39.2 (3.5)	39.2 (3.6)
	Missing	1.9	
Cognitive impairment (score range: 0–9)		7.9 (1.2)	7.9 (1.2)
	Missing	6.1	
Loneliness	Often	4.2	4.5
	Sometimes	10.3	10.6
	Rarely	83.7	84.9
	Missing	1.8	
Father’s highest educational level	No formal education	1.7	2.6
	Junior high school graduation	55.4	70.8
	High school graduation	11.6	14.8
	University graduation	9.3	11.8
	Missing	22.1	
Mother’s highest educational level	No formal education	1.6	2.7
	Junior high school graduation	56.3	69.9
	High school graduation	19.3	23.6
	University graduation	3.1	3.8
	Missing	19.6	
Financial disadvantage	≤18 years old (score range: 1–4)	2.6 (1.0)	2.6 (1.0)
	Missing	2.3	
	25–35 years old (score range: 1–4)	3.0 (0.8)	3.0 (0.8)
	Missing	1.1	
	35–50 years old (score range: 1–4)	3.1 (0.8)	3.1 (0.8)
	Missing	1.1	
	Current age (score range: 1–4)	2.9 (0.8)	2.9 (0.8)
	Missing	2.9	
Depressive mood	≥9 in the CES-D-8 score	16.8	17.1
	Missing	3.0	

CES-D-8, The 8-item Center for Epidemiologic Studies Depression.

Values represent mean (standard deviation) or %.

**Figure 1.**
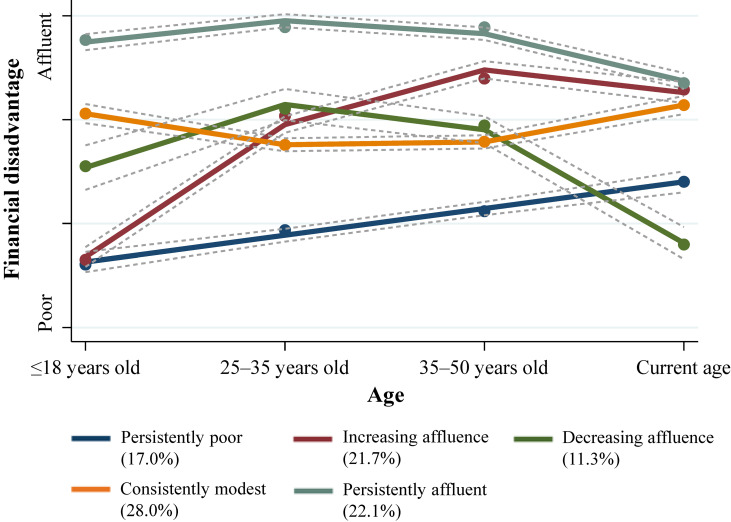
Trajectories of financial disadvantage over the life course

Figure 1 shows the trajectories of the life-course financial disadvantages derived from the GBMM, and Table 2 shows the intercept and linear and quadratic slopes of each trajectory. The trajectories in Figure 1 were derived from only one of the 20 imputed datasets because the trajectory patterns from each imputed dataset were similar. Consequently, five distinct trajectories were identified as the best-fitting model: “persistently affluent” (22.1% of the sample [*n* = 281]; quadratic pattern), “increasing affluence” (21.7% of the sample [*n* = 317]; quadratic pattern), “consistently modest” (28.0% of the sample [*n* = 369]; quadratic pattern), “decreasing affluence” (11.3% of the sample [*n* = 125]; quadratic pattern), and “persistently poor” (17.0% of the sample [*n* = 232]; linear pattern). eTable 1 presents the AIC and BIC scores based on the number of distinct trajectories.

**Table 2.** Estimates of growth curve parameters for distinct trajectories of life-course financial disadvantage

	Intercept	Linear slope	Quadratic slope	Group membership
		
	Estimate (95% CI)	Estimate (95% CI)	Estimate (95% CI)	
Persistently affluent	3.21 (3.00–3.42)	0.70 (0.51–0.89)	−0.17 (−0.20 to −0.13)	22.1%
Increasing affluence	−0.31 (−0.59 to −0.04)	2.37 (2.12–2.62)	−0.37 (−0.42 to −0.32)	21.7%
Consistently modest	3.71 (3.46–3.96)	−0.79 (−1.02 to −0.57)	0.16 (0.12–0.21)	28.0%
Decreasing affluence	1.11 (0.56–1.65)	1.84 (1.38–2.30)	−0.42 (−0.51 to −0.33)	11.3%
Persistently poor	1.35 (1.20–1.49)	0.27 (0.21–0.32)	—	17.0%

CI, confidence interval.

Table 3 presents a comparison of the characteristics of the five trajectory subgroups. The subgroups of “increasing affluence” and “persistently poor” had the highest mean age. Approximately 60% of the “increasing affluence” and “decreasing affluence” subgroups and roughly 40% of the “persistently affluent” and “consistently modest” were men.

**Table 3.** Participants’ characteristics by trajectories of life-course financial disadvantage

		Persistently affluent (*n* = 281)	Increasing affluence (*n* = 317)	Consistently modest (*n* = 369)	Decreasing affluence (*n* = 125)	Persistently poor (*n* = 232)	*P*-value
Age, years		70.3 (7.6)	72.4 (7.4)	71.7 (8.1)	68.2 (6.4)	72.2 (7.8)	<0.001^a^
Sex	Men	42.6	57.9	43.8	58.9	48.1	<0.001^b^
Marital status	Married	76.3	78.7	71.7	76.8	67.9	0.255^b^
	Never married	2.8	2.4	2.7	3.2	2.2	
	Divorced	4.3	2.9	4.5	4.8	7.0	
	Widowed	16.6	15.9	21.0	15.2	22.9	
Living alone		11.4	10.7	14.8	15.0	12.8	<0.001^b^
Employment status	Current working	34.2	30.7	38.3	46.2	36.9	<0.001^b^
Year of education	≤9 years	17.6	39.2	29.9	24.6	45.4	<0.001^c^
	10–12 years	42.6	37.7	46.9	49.5	36.2	
	≥13 years	39.9	23.1	23.2	25.9	18.4	
Annual individual/couple income	≤2.9 million yen	37.9	42.4	51.6	53.3	65.7	<0.001^c^
	3.0–3.9 million yen	24.5	26.6	24.2	24.1	16.0	
	≥4.0 million yen	37.6	31.0	24.2	22.5	18.2	
Smoking habit	Currently smoking	12.4	13.8	12.0	23.4	15.7	<0.001^b^
Exercise habit	Often/sometimes	70.2	70.0	63.4	61.6	57.6	<0.001^b^
Hypertension		38.0	44.3	42.7	38.3	44.5	0.409^b^
Heart disease		14.0	13.8	9.0	12.9	11.6	0.239^b^
Cerebrovascular disease		5.4	6.3	4.6	7.1	5.7	0.731^b^
Cancer		5.4	5.5	5.1	2.4	6.4	0.554^b^
Diabetes mellitus		10.7	14.5	11.3	13.8	13.6	0.554^b^
Functional capacity (score range: 11–55)		39.3 (3.1)	39.2 (3.8)	39.3 (3.0)	39.1 (4.1)	38.8 (4.2)	0.546^a^
Cognitive impairment (score range: 0–9)		8.0 (1.1)	8.0 (1.2)	7.9 (1.3)	8.0 (1.0)	7.7 (1.3)	0.038^a^
Loneliness	Often	3.3	3.2	4.5	6.7	6.4	<0.001^c^
	Sometimes	7.9	8.2	8.5	17.5	16.9	
	Rarely	88.8	88.7	87.0	75.8	76.6	
Father’s highest educational level	No formal education	1.7	3.0	2.1	2.3	4.2	<0.001^c^
	Junior high school graduation	59.8	76.1	70.9	71.2	76.2	
	High school graduation	20.4	11.5	16.9	13.1	10.1	
	University graduation	18.1	9.3	10.1	13.4	9.5	
Mother’s highest educational level	No formal education	1.2	3.2	2.0	2.4	5.4	<0.001^c^
	Junior high school graduation	61.6	73.3	69.3	70.4	75.9	
	High school graduation	30.9	20.7	24.7	25.8	15.7	
	University graduation	6.3	2.8	4.0	1.4	3.0	

CES-D-8, The 8-item Center for Epidemiologic Studies Depression.

Values represent mean (standard deviation) or %.

^a^One-way analysis of variance.

^b^Chi-square test.

^c^Kruskal–Wallis test.

Finally, Table 4 indicates the association between the trajectories of life-course financial disadvantages and depressive mood. Logistic regression analysis showed that people in the “persistently affluent” and “increasing affluence” subgroups were less likely to have depressive mood than those in the “persistently poor” subgroup after adjusting for age and sex in model 1 (OR 0.58; 95% CI, 0.37–0.92 for “persistently affluent” and OR 0.47; 95% CI, 0.30–0.75 for “increasing affluence”). After adjusting for other sociodemographic characteristics, health behaviors, health conditions, and parental educational attainment in model 2, the association between “increasing affluence” and depressive mood remained (OR 0.60; 95% CI, 0.36–0.98), although that between “persistently affluent” and depressive mood became nonsignificant (OR 0.72; 95% CI, 0.43–1.19). The predicted probabilities of depressive mood in the “increasing affluence” and “persistently affluent” subgroups were 12.5% and 15.8%, while that in the “persistently poor” subgroup was 24.1%.

**Table 4.** Association between trajectories of life-course financial disadvantage and depressive mood

	Model 1	Model 2	
	
	OR (95% CI)	OR (95% CI)	Predicted probabilities
Persistently affluent	0.58 (0.37–0.92)	0.72 (0.43–1.19)	15.8%
Increasing affluence	0.47 (0.30–0.75)	0.60 (0.36–0.98)	12.5%
Consistently modest	0.68 (0.44–1.04)	0.84 (0.53–1.32)	18.3%
Decreasing affluence	1.02 (0.59–1.77)	0.93 (0.51–1.69)	21.6%
Persistently poor	Ref.	Ref.	24.1%

CI, confidence interval; OR, odds ratio.

Model 1: Adjusted for age and sex.

Model 2: Additionally adjusted for marital status, living alone, employment status, years of education, annual individual/couple income, smoking habit, exercise habit, hypertension, heart disease, cerebrovascular disease, cancer, diabetes mellitus, functional capacity, cognitive impairment, loneliness, father’s highest educational level, and mother’s highest educational level.

We additionally conducted the stratified analysis by age group (60–74 years and ≥75 years) and sex (eTable 2). In the age-stratified analysis, the results were similar to those of the total analysis, although the estimates did not reach statistical significance. In contrast, the sex-stratified analysis showed that the association between “increasing affluence” and depressive mood was more prominent in women (OR 0.48; 95% CI, 0.24–0.94) than in men (OR 0.79; 95% CI, 0.35–1.76).

## DISCUSSION

This study highlights the importance of lifetime trajectories of financial disadvantage in shaping the mental health of older adults. Earlier studies used an a priori classification of SES trajectories. Trajectory analysis enables identification of naturally occurring patterns in longitudinal data without relying on predefined categories, thereby minimizing researcher bias. This approach provides a more data-driven and nuanced understanding of the heterogeneity within populations, uncovering latent subgroups that might otherwise be overlooked.

While similar findings regarding depressive mood were reported previously using the same dataset,^11^ the analysis relied on a priori classifications of financial trajectories based on combinations of financial strain at multiple time points; upward mobility was associated with fewer depressive moods, whereas downward mobility was linked to more. Participants with upward mobility following downward mobility had significantly more depressive mood than those without financial strain. However, these prespecified categories may not fully capture the diversity of financial trajectories. In contrast, our study employed GBMM to empirically identify patterns of financial disadvantage over time, offering a more nuanced and data-driven understanding of its association with depressive mood.

Our findings suggest that overcoming financial disadvantages, rather than maintaining a lifelong wealthy status, contributes to lower depressive mood in old age. Specifically, individuals in the “increasing affluence” subgroup exhibited significantly lower probabilities of depressive mood than those in the “persistently poor” subgroup, even after adjusting for sociodemographic characteristics, health behaviors, health conditions, and parental education. These results emphasize that upward financial mobility has a protective effect on mental health. Previous studies have reported that current financial strain, independent of earlier life stages, such as childhood, is strongly associated with present depressive mood.^11^^,^^13^^,^^14^ This suggests that contemporary socioeconomic disadvantage plays a critical role in mental health. However, our findings indicate that an individual’s financial trajectory, particularly the path to current affluence, may be even more significant.

Previous studies have shown that upward socioeconomic mobility mitigates the adverse effects of childhood disadvantages by reducing chronic stress and fostering resilience.^31^^,^^32^ Although upward mobility has clear benefits, it is not without challenges. For some individuals, transitioning to higher SES may lead to feelings of alienation or discrimination from a new social group, potentially affecting physical and mental health.^33^ However, longitudinal studies suggest that individuals who achieve upward mobility experience lower stress levels than those who experience downward mobility.^34^ Successful experiences throughout the life course could enhance stress resistance.^35^ Cognitive enrichment associated with upward mobility may also play a role in mental health by counteracting stress-induced reductions in hippocampal volume.^36^^–^^38^ This aligns with findings indicating that growth mindsets and lifelong learning, which are often observed in upwardly mobile individuals, enhance cognitive function and psychological well-being.

Furthermore, evidence suggests that the health benefits of upward socioeconomic mobility may differ by sex. A stratified analysis from a large-scale Japanese cohort study found that an upward socioeconomic trajectory was significantly associated with a reduced risk of mortality only among women, and that this association was attenuated after adjusting for depressive mood.^39^ This finding indicates that lower levels of depressive mood may mediate the protective effects of upward mobility in women. One possible explanation is that women may be more sensitive to improvements in social and psychological resources accompanying upward mobility, such as enhanced social support and greater autonomy in managing role-related demands. These factors could foster emotional resilience and buffer against psychological distress, especially for those who have experienced adversity in early life.

In the Japanese context, participants who experienced financial hardship during childhood likely faced significant adversity, given the postwar period of economic disparity.^40^ However, rapid economic growth in Japan (mid-1950s to mid-1970s) during the participants’ youth and middle age provided opportunities for upward mobility, which may have contributed to improved mental health in later years. Notably, those who reported poverty in this era likely faced severe hardships. Our findings align with the broader literature suggesting that both childhood and adult SES are critical for health outcomes and that interventions targeting social mobility could mitigate the long-term effects of early life disadvantages.^41^^,^^42^

The subgroups of “persistently poor” and “decreasing affluence” had similar high probabilities of depressive mood. This finding is consistent with previous studies.^10^^,^^11^^,^^14^^,^^22^ Chronic financial strain may create problems within family and social relationships and lead to a decline in personal resources, such as self-esteem and social support, compounding its effects over time. One reason why chronic financial strain appears impactful is its persistent nature, which can lead to the accumulation of additional stressful experiences over time. Moreover, declining financial comfort can also be stressful as they are unable to maintain the same standard of living as before. These are associated with an elevated allostatic load, the physiological burden imposed by chronic stress that hinders the body’s ability to recover from stress, thereby exacerbating health outcomes,^43^^,^^44^ particularly mental health issues.^45^ Allostatic load is a key mechanism linking persistent financial disadvantages to adverse health outcomes, as it reflects the wear and tear of biological systems due to prolonged stress exposure. Therefore, those in the “persistently poor” and “decreasing affluence” subgroups were likely to have a depressive mood.

Based on our findings, policies that support upward financial mobility across the life course may help reduce depressive mood in later life. Such interventions could include financial assistance for education and job training in early adulthood, employment support programs, and accessible social safety nets throughout life. Additionally, programs aimed at promoting financial literacy, re-employment opportunities for older adults, and targeted support for socioeconomically disadvantaged individuals may be beneficial.

This study had some limitations. First, this was a cross-sectional study, and life-course financial disadvantage was assessed based on participants’ retrospective reports, which may introduce misclassification of financial disadvantages and their trajectories. Second, because detailed information on SES in childhood and adulthood and other information was limited, the possible pathway between the life-course financial disadvantage and depressive mood in later life is unclear. For example, we did not measure history of disease possession, including past depression history, and the use of medication, including antidepressant intake. These factors might be confounders in the relationship between trajectories of life-course financial disadvantage and depressive mood. Further studies are required to examine the mediators of this association. Third, the generalizability of the findings may be limited by the specific historical context of the study participants. Many experienced their working years during Japan’s rapid economic growth period, when stable employment and seniority-based systems made upward mobility more achievable. In contrast, today’s younger generations face more unstable job markets and limited income growth. Therefore, the relationship between financial trajectories and mental health observed in this cohort may not fully apply to current or future populations.

### Conclusions

This study emphasizes the critical role that lifetime financial trajectories play in influencing older adults’ mental health. The findings underscore the importance of social mobility for health. This suggests that while childhood is critical, mental health conditions in old age are not inevitably programmed into childhood. Upward mobility later in life can partially alleviate worse mental health conditions. We also highlighted the importance of policies aimed at reducing socioeconomic disparities and promoting upward mobility, which could improve mental health outcomes and enhance resilience in aging populations.

## SUPPLEMENTARY MATERIAL

eTable 1. The Akaike Information Criterion and Bayesian Information Criterion scores by the number of trajectories

eTable 2. Stratified analyses in the association between trajectories of life-course financial disadvantage and depressive mood by age group and sex
