# Supplementary material for: Trajectories of Life Course Financial Disadvantage and Depressive Mood: Results From the National Survey of the Japanese Elderly
Source: J Epidemiol. 2026 Apr 5;36(4):132–9. doi: 10.2188/jea.JE20250159 (PMC12975770; doi:10.2188/jea.JE20250159)
Supplement: Supplementary file 1 [file je-36-132-s001.pdf]

eTable 1. The Akaike Information Criterion and Bayesian Information Criterion scores by the number of trajectories

		AIC	BIC
Number of trajectories	2	12325.07	12465.15
	3	11978.66	12191.38
	4	11919.12	12204.49
	5	11884.71	12242.71
	6	11877.64	12308.28
	7	11875.22	12378.5
	8	11882.31	12458.23

AIC, Akaike Information Criterion; BIC, Bayesian Information Criterion.

eTable 2. Stratified analyses in the association between trajectories of life-course financial disadvantage and depressive mood by age group and sex

	60–74 years	≥75 years	Men	Women
	OR (95% CI)[a]	OR (95% CI)[a]	OR (95% CI)[b]	OR (95% CI)[b]
Persistently affluent	0.81 (0.42–1.60)	0.68 (0.30–1.53)	0.88 (0.35–2.17)	0.64 (0.34–1.19)
Increasing affluence	0.62 (0.31–1.24)	0.60 (0.27–1.30)	0.79 (0.35–1.76)	0.48 (0.24–0.94)
Consistently modest	0.73 (0.38–1.40)	0.99 (0.49–2.01)	0.82 (0.37–1.85)	0.82 (0.46–1.45)
Decreasing affluence	1.10 (0.53–2.27)	0.75 (0.21–2.67)	2.16 (0.91–5.16)	0.43 (0.17–1.09)
Persistently poor	Ref.	Ref.	Ref.	Ref.

CI, confidence interval; OR, odds ratio.

[a] Adjusted for sex, marital status, living alone, employment status, years of education, annual individual/couple income, smoking habit, exercise habit, hypertension, heart disease, cerebrovascular disease, cancer, diabetes mellitus, functional capacity, cognitive impairment, loneliness, father's highest educational level, and mother's highest educational level.

[b] Adjusted for age, marital status, living alone, employment status, years of education, annual individual/couple income, smoking habit, exercise habit, hypertension, heart disease, cerebrovascular disease, cancer, diabetes mellitus, functional capacity, cognitive impairment, loneliness, father's highest educational level, and mother's highest educational level.
